# Effect of simulated root canal morphology and post space depth on fracture performance of glass fiber-reinforced post-core buildups

**DOI:** 10.1186/s12903-025-07424-x

**Published:** 2025-12-17

**Authors:** Salma Magdy Wahby, Khaled Aly Nour, Omaima Hassan Ghallab, Khaled Mohamed Adel

**Affiliations:** 1 Conservative Dentistry Department, Faculty of Dentistry, Ain Shams University, Cairo, Egypt; 2 Conservative Dentistry Department, Faculty of Oral and Dental Medicine, Egyptian Russian University, Cairo, Egypt

**Keywords:** Root canal morphology, Fracture strength, Glass fiber-reinforced composite, Post space depth.

## Abstract

**Objectives:**

This *in vitro* study evaluated the effect of simulated root canal morphology, post space depth (PSD), and type of glass fiber-reinforced composite (GFRC) on the fracture strength and failure mode of post-core buildups.

**Materials and methods:**

A total of 168 3D-printed maxillary premolar root models were allocated into 24 groups (*n* = 7) based on simulated canal morphology (single-circle, oval, double-circle), PSD (4 mm, 6 mm), and GFRC type (everStick, everX-flow, everX-posterior), with Itena post as the control. A standardized adhesive protocol was used, followed by GFRC application and dual-cure core buildup. Fracture strength was tested under a 45° compressive load. Failure modes were categorized as favorable (type I, II, III) or unfavorable (type IV). Data were analyzed using ANOVA with Tukey’s post hoc test, independent t-tests, and chi-square tests (α = 0.05).

**Results:**

GFRC type significantly affected fracture strength (*p* < 0.001), whereas neither simulated canal morphology nor PSD showed a significant effect. At 4 mm PSD, everX-flow exhibited significantly lower fracture strength than Itena and everStick posts in single-circle and oval canal models (*p* < 0.05). At 6 mm PSD, everX-flow recorded the lowest fracture strength in single-circle canal models (*p* < 0.05). Failure mode was significantly influenced by PSD (*p* = 0.01) and GFRC type (*p* < 0.001). A 6 mm PSD was associated with a higher percentage of each favorable failure type compared to 4 mm PSD. EverStick and Itena posts showed the highest percentage of favorable failures.

**Conclusions:**

The intra-canal reinforcing material is the primary determinant of the fracture performance of post-core buildups. The mechanical reinforcement materials of everStick and everX-posterior posts but not everX-flow are comparable to that of Itena post.

**Clinical relevance:**

Everstick and everX-posterior can replace Itena posts as safe reinforcing materials in single-canaled premolars.

## Introduction

The long-term structural integrity and biomechanical performance of endodontically treated teeth (ETT) are often compromised [1], particularly in maxillary premolars [2]. Consequently, intracanal posts and core buildups are employed to reinforce the remaining tooth structure and enhance fracture resistance against masticatory forces [2]. Several factors, including root canal morphology, restorative procedures, and materials, have a significant influence on the fracture strength of ETT [1, 3].

The root canal morphology of maxillary premolars is a crucial determinant of the biomechanical performance of post-endodontic restorations [4]. Compared to circular canals, oval canals generate non-uniform stress distributions during functional loading, particularly at the post-dentin interface [5]. Restoring oval canals with prefabricated posts designed for round canal geometries often results in poor adaptation, residual gaps, stress concentration points, and compromised reinforcement [6].

Increased post length has traditionally been associated with improved retention and favorable stress distribution [2]. However, significant dentin removal during post space preparation undermines the resilience of tooth structure and increases susceptibility to vertical root fractures [7]. The risk is particularly pronounced in anatomically narrow or bifurcated canals [8, 9]. Although numerous studies have assessed the role of post length in improving the retention and load-bearing capacity of ETT, limited attention has been paid to how canal anatomy should guide the determination of optimal post length and design [9, 10].

To address the risks associated with excessive dentin removal during post space preparation, glass fiber-reinforced composite (GFRC) systems have emerged as a promising alternative to traditional post-core systems [11]. GFRC’s improved physical and mechanical properties promote homogeneous stress distribution and limit crack propagation, thereby reducing the risk of catastrophic failure [11, 12]. The clinical performance of prefabricated posts has shown inconsistent fracture resistance and long-term outcomes, largely due to their poor adaptation to root canal walls, which compromises core homogeneity and creates stress concentration zones [10, 13]. To overcome these limitations, the bio-block technique was introduced, in which the coronal cavity and root canal are filled with GFRC to create a uniform post-core system that resists crack propagation [12].

While several studies have independently assessed the influence of root canal morphology [14], post length [7], or core material [12], on the fracture resistance of ETT, their combined biomechanical impact remains largely unexplored. This knowledge gap is critical, as these factors are not mutually exclusive in clinical practice but coexist and interact synergistically, influencing treatment outcomes. The interplay between root canal configuration, intracanal post space depth, and GFRC type is underexplored. A comprehensive understanding of these interdependencies is essential to optimize restorative strategies for structurally compromised teeth.

Root canal morphology was included as an independent variable to account for the influence of anatomical configurations on stress distribution and adaptation of different reinforcing materials. Since oval and multi-canal morphologies are frequently encountered clinically, assessing how both prefabricated and moldable fiber-reinforced systems interact with variable canal geometries enhances the translational relevance of the findings.

Therefore, the present study aims to evaluate the effect of simulated root canal morphology, post space depth (PSD) and GFRC type on the fracture strength and failure mode of post-core buildups. The first null hypothesis states that simulated root canal morphology, PSD and GFRC type do not affect the fracture strength of post-core buildups. The second null hypothesis states that simulated root canal morphology, PSD, and GFRC type have no effect on the failure mode of post-core buildups.

## Materials and methods

This study used one prefabricated glass fiber post as a control (Itena post) and three types of GFRC systems (everStick post, everX-floe, and everX-posterior). A universal adhesive and a dual-cured core buildup composite were also employed. Details of all materials including chemical composition, lot numbers, and manufacturers, are presented in Table 1.

**Table 1:** Material, chemical composition, lot #, and manufacturers

Material	Chemical Composition	Lot #	Manufacturers
GRADIA™ CORE (Dual-cured core build-up composite) Shade: universal	Methacrylic acid ester 20–30 wt%, fluoro-alumino-silicate glass 70–75 wt%, silicon dioxide 1–5 wt%.	2,307,041	GC Corporation, Tokyo, Japan
All-Bond Universal (Ultra-mild universal adhesive, pH 3.2).	Bis-GMA, 10-MDP, 2-HEMA, ethanol, water, and photo-initiator.	2,300,010,910	Bisco, Schaumburg, Illinois, USA.
DentoClic glass fiber post. (Translucent 1.40 mm diameter- Red).	80% unidirectional, parallel glass fiber embedded in 20% epoxy-resin.	57,597	Itena Clinical, Villepinte, France.
everStick™ POST 1.5. Individually formable glass fiber root canal posts.	Unidirectional bundle of silanated E-glass fiber impregnated with Bis-GMA and PMMA.	230,413 A	GC Europe, Finland
everX Flow™ Short fiber reinforced flowable composite for dentin replacement. Bulk Shade	Bis-EMA, TEGDMA, UDMA, short E- glass fiber, barium glass. Fiber loading: 70 wt%	2,001,241	GC Corporation, Tokyo, Japan
everX Posterior ^TM^ Fiber reinforced composite for dentin replacement.	Bis-GMA, TEGDMA, PMMA, Short E-glass fber, barium glass and silicon dioxide. Fiber loading: 74.2 wt	2,204,061	GC Corporation, Tokyo, Japan

*Bis-GMA* Bisphenol A-glycidyl methacrylate, *UDMA* Urethane dimethacrylate, *TEGDMA* Triethylene glycol dimethacrylate, *MDP* Methacryloyloxydecyl dihydrogen phosphate, *HEMA* Hydroxylethyl methacrylate, *Bis*-*EMA* Ethoxylated bisphenol a glycol dimethacrylate.

The Itena post (DentoClic, Itena Clinical, Villepinte, France) served as the control prefabricated glass fiber post

### Sample size calculation

The required sample size was determined using G*Power version 3.1.9.7 software (G*power; Heinrich Heine University, Düsseldorf, Germany), based on the results of a comprehensive pilot study. The effect size was Cohen’s f = 0.30 [15]. With 24 experimental groups, a significance level (α = 0.05) and power (1-β = 0.90), the analysis indicated a total sample size of 168 specimens (*n* = 7 in each group).

### Study design

This *in vitro* experimental study aimed to evaluate the effect of simulated root canal morphology (single-circle, oval, and double-circle), PSD (4 mm and 6 mm), and GFRC type (everStick post, everX-flow, everX-posterior) on the fracture strength and failure mode of post-core buildups. The prefabricated glass fiber post (Itena post) served as the control. Fracture strength was the primary outcome, measured in Newtons (N) followed by failure mode analysis.

### Specimen preparation

Standardized root models, and core buildups were designed and fabricated with meticulous attention to detail, ensuring consistency across all specimens and adherence to manufacturers instructions.

#### a. 3D digital designing and printing

To minimize confounding effect of anatomical variability in natural teeth-which can obscure the influence of experimental factors and limit reproducibility [16]-standardized 3D-printed root models were fabricated using cone-beam computer tomography (CBCT) data. A total of 50 CBCT scans of 120 maxillary premolars were collected in accordance with the ethical guidelines from the Research Ethics Committee of the Faculty of Dentistry, Ain Shams University (approval no. FDASU-RecED032150). The CBCT scans, in addition to anatomical literature [17–19], were analyzed to guide the design of simulated root canal models and ensure clinical relevance while eliminating inter-specimen variability [20].

Two root segment heights (4 mm and 6 mm), simulating the cervical and middle root thirds, were designed using computer-aided design software (AutoCAD 2022, version 24.1; Autodesk Inc., San Francisco, California, USA), reflecting stress-prone regions in maxillary premolars under oblique forces [21]. These two lengths were specifically selected to highlight the reinforced effect using the minimal reported anchorage depths in the root canal [12].Each model had total dimensions of 8.02 ± 0.89 mm buccopalatal, 4.14 ± 1.53 mm mesiodistal, and a height of either 4–6 mm, according to the assigned group.

**Fig. 1:**
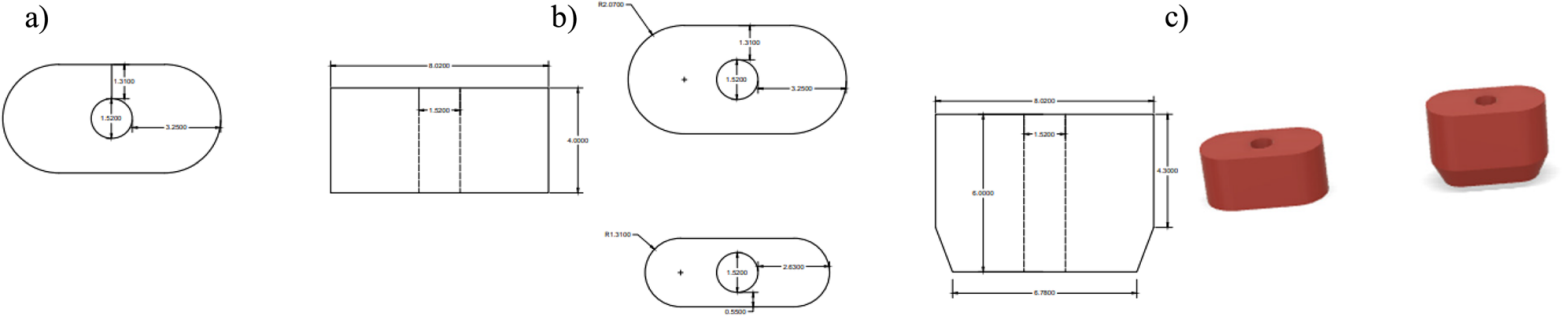
**a** Schematic design of the 4 mm single central circular canal dimensions, (**b**) Schematic design of the 6 mm single central circular canal dimensions, and (**c**) corresponding 3D designs

**Fig. 2:**
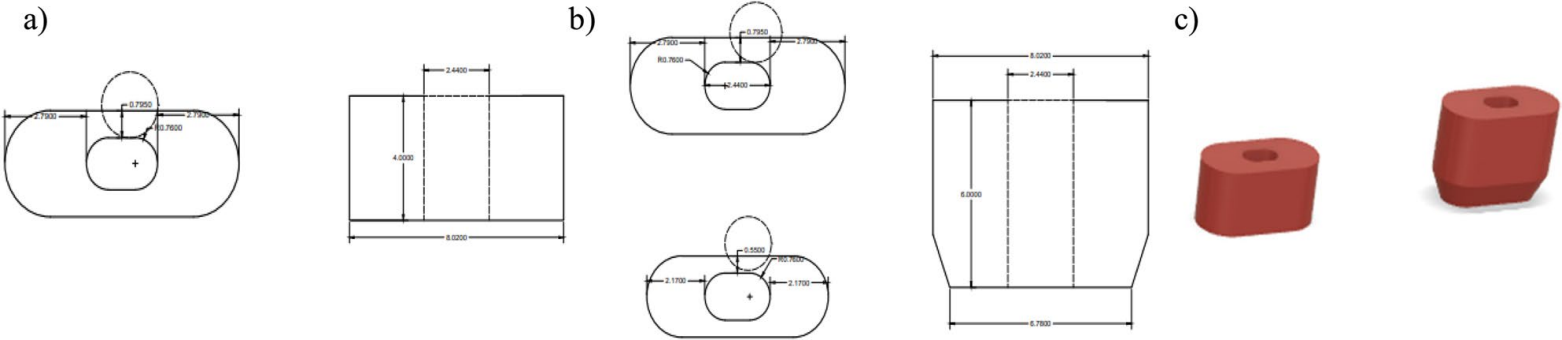
**a** Schematic design of the 4 mm single oval canal dimensions, (**b**) Schematic design of the 6 mm single oval canal dimensions, and (**c**) corresponding 3D designs

**Fig. 3:**
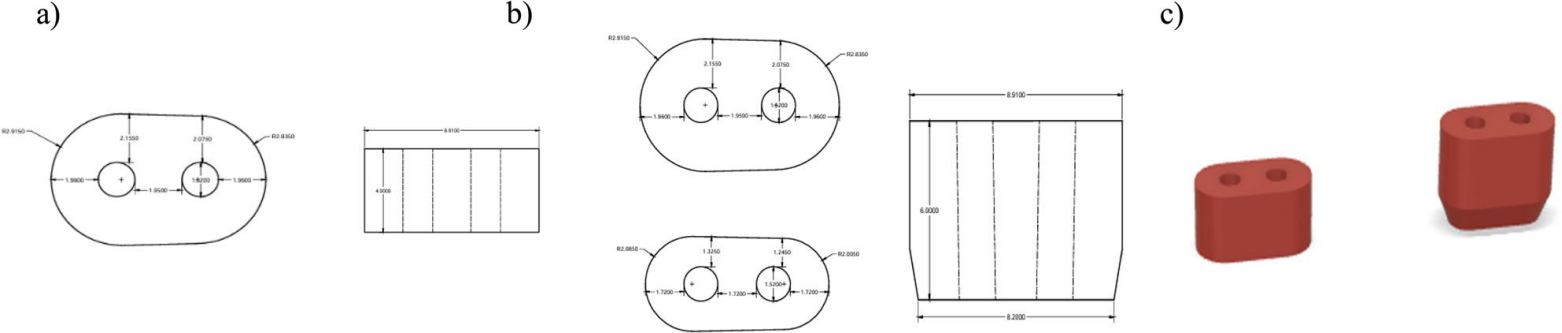
**a** Schematic design of the 4 mm double circular canal dimensions, (**b**) Schematic design of the 6 mm double circular canal dimensions, and (**c**) corresponding 3D design

Each root model featured one of three distinct simulated canal morphologies: single-circle (1.52 mm in diameter), oval (long/short ratio>2) [22], and double-circle (diameter = 1.52 mm per canal), representing common anatomical variations in maxillary premolars (Figs. 1, 2 and 3). Rigid 10 K resin (Formlabs; Formlabs Inc.,Somerville, Massachusetts, USA) was selected for mold printing due to its modulus of elasticity (~11 GPa) [23], which approximates the lower range of natural dentin (~12–20 GPa) [24, 25], using a Form 3 stereolithography (SLA) 3D printer (Formlabs; Formlabs Inc.,Somerville, Massachusetts, USA), at a 50 µm layer thickness and 85 µm laser spot size to ensure highly detailed outcomes [26]. This mechanical simulation facilitated physiologically relevant stress distribution during fracture testing.

**Fig. 4:**
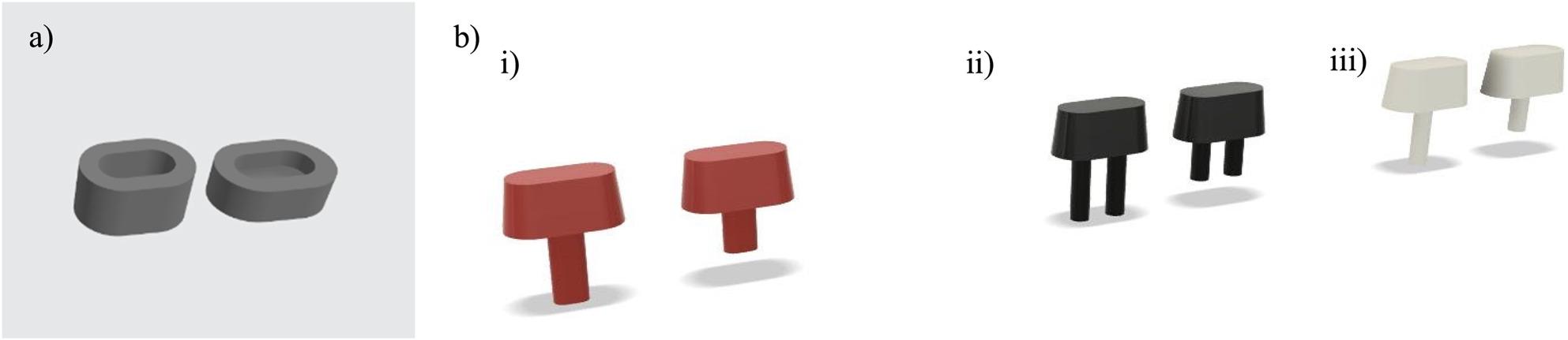
**a** 3D models of the mold base of both 6 mm and 4 mm molds respectively, **b** 3D design of the core, (i) 6 mm and 4 mm core for oval canal analogues, (ii) 6 mm and 4 mm core for double-circle canal analogues, and (iii) 6 mm and 4 mm core for single-circle canal analogues

A 2 mm-thickness mold base and a non-anatomical core of 4 mm height with the same dimensions as the root models, were also designed (Figure 4) and printed in Tough 1500 resin, (Formlabs; Formlabs Inc., Somerville, Massachusetts, USA) using a Form 2 SLA 3D printer (Formlabs; Formlabs Inc., Somerville, Massachusetts, USA), at a 100 µm layer thickness and 140 µm laser spot size. The non-anatomical core design was used to ensure consistent geometry, minimize variability from natural anatomy, and isolate the mechanical performance of the reinforced core in relation to fracture strength. The standardized 4 mm core height provided enough structure for the load application as close as possible to the reinforcement material (post/orifice region), so that the results would predominantly reflect it mechanical response rather than that of the mold or core materials. During specimen preparation, the mold base aided in mold handling, while the core model served as a positive replica for core fabrication. 

Following the manufacturer’s recommendations, all printed models underwent standardized post-processing protocol, including a 20-minute wash in 99% isopropyl alcohol to remove uncured resin, followed by air-drying. Models were then post-cured at 70 °C for 60 min using a 405 nm UV light post-processing unit (Form Cure, Formlabs Inc., Somerville, Massachusetts, USA) to achieve full mechanical properties and dimensional stability.

To visually differentiate the root molds from the core buildups without affecting the mold’s mechanical performance, root molds were coated with a thin layer of matte grey acrylic paint after surface preparation using light sandblasting and priming. All printed models were later visually inspected for printing defects, surface irregularities, or dimensional inaccuracies, verified using a digital caliper (TOTAL; Total tools Co Limited, INOX, China).

#### b. Reinforcement and core buildup

All three printed parts (core, root mold, and mold base) were assembled. For standardization, a soft, clear vacuum sheet of 0.8 mm thickness (SPLINT; Longma Machinery and Electronics Co. LTD, Shangyu China), followed by a hard 1 mm sheet, were used to create clear indices for core fabrication [27, 28]. Excess material was trimmed, and a hole was created above the middle of the occlusal surface using a low-speed round bur (ISO 012) under water irrigation to serve as a channel for restorative material insertion.

The root mold was disassembled, and All-Bond Universal adhesive was applied and agitated in the simulated canal and on the occlusal surface in two coats (15 s each), followed by air-drying (5 s) and light-curing (10 s on each mold end) using a fully charged LED unit (SDI Radii Plus; SDI Limited, Bayswater, Victoria, Australia) with an output of 1500 mW/cm² and a wavelength of 440–480 nm, directed perpendicularly on the adhesive layer once per root mold side [29].

Each 3D-printed mold representing a specific simulated root canal morphology and PSD was allocated to one of four groups according to the reinforcing material. In the double-circle canal analogues, both canals received the same reinforcing material. Before reinforcement application, mold stabilization was carried using a custom-made metal jig with tightening screws that can hold the mold in place until reinforcement protocol is completed. Reinforcing material was placed into the canal analogues and extended 3 mm coronally.

### Control group

**Fig. 5:**
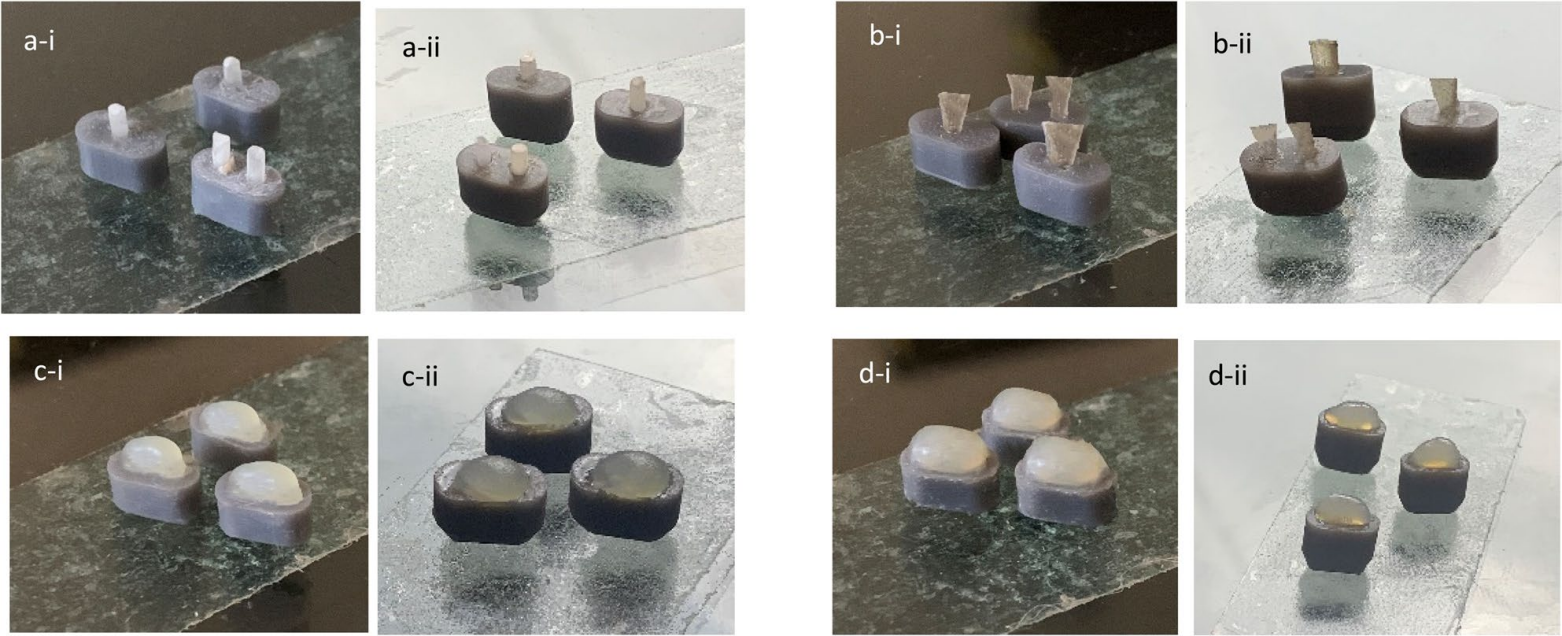
Representative images of the reinforcement treatment for the molds of three simulated root canal morphologies at two post space depth (4 mm and 6 mm). **a** Itena post: (**a**-i) 4 mm PSD, (**a**-ii) 6 mm PSD; (**b**) everStick post: (**b**-i) 4 mm PSD, (**b**-ii) 6 mm PSD; (**c**) everX-flow: (**c**-i) 4 mm PSD, (**c**-ii) 6 mm PSD; (**d**) everX posterior: (**d**-i) 4 mm PSD, (**d**-ii) 6 mm PSD

Specimens in this group received the Itena post (Fig. 5a-i and a-ii). The post was measured using a periodontal probe (Lascod Zeffiro, Florence, Italy) and trimmed to the required length using a high-speed FG ultra-fine tapered with round end diamond bur no. 846RC (Meisinger; Hager & Meisinger GmbH, Neuss, Germany) under copious water irrigation. The trimmed post was then cleaned with 70% ethyl alcohol, treated with silane (Silano silane coupling agent; Maquira Industry of Dental Products Ltd, Londrina, Brazil), and to dry completely to enhance chemical bonding. Subsequently, Gradia core was injected into the simulated canal to act as a dual-cure resin cement, followed by insertion of the trimmed Itena post. Excess cement was carefully removed using a clean bond brush, and the mold was light-cured for 10 s from both coronal and apical mold sides to ensure adequate depth of cure.

#### Group 1

This group received everStick post as the reinforcement material. The everStick post was pre-cut to the required length using sharp scissors according to the manufacturer’s instructions. For circular canal analogues, the adaptation of the everStick post was slightly trimmed to achieve the standardized diameter of 1.4 mm. Dimensions were verified using a digital caliper, and passive seating of the post within the canal analogue was confirmed under 3.5× magnification (UNIVET Loupes; UNIVET Group, Rezzato, Italy). After mold stabilization and adhesive application, Gradia core was used solely as a dual-cure resin cement to lute the everStick post within the canal analogue. Excess material was removed, and the mold was light-cured for 10 s from both coronal and apical mold sides, following the manufacturer’s instructions (Fig. 5b-i and b-ii).

Cementation was performed for the control group and group 1, to guarantee retention and stress transfer along interfaces, reflecting their clinical application, whereas moldable GFRC materials were directly inserted without a luting agent, as they are designed to adapt and polymerize in situ as monoblock systems. This approach was maintained intentionally to preserve clinical relevance rather than introduce methodological inconsistency. Gradia core exhibits a curing depth of 2.5 mm under 10 s of light exposure at > 1200 mW/cm^2^and complete polymerization through a 5-minute self-cure mechanism.

#### Group 2

After mold stabilization and adhesive application, the canal analogues were filled in a single incremental injection with everX-flow and light-cured. Light-curing protocol followed the manufacturer’s validated recommendations: 10 s for coronal and apical mold side each using a LED curing unit with an output of 1500 mW/cm^2^, resulting in a total irradiation of 20 s per specimen. EverX-flow (bulk shade) achieves a curing depth of 5.5 mm after 10 s of light exposure at > 1200 mW/cm^2^. A 3 mm coronal everX-flow extension was made, and light-cured for 10 s (Fig. 5c-i and c-ii).

#### Group 3

After mold stabilization and adhesive application, the canal analogues were filled in a single incremental packing with everX-posterior using a small, smooth-end condenser. The same light-curing protocol as group 2 was adopted. EverX-posterior achieves a curing depth of 4 mm after 10 s of light exposure at > 1200 mW/cm^2^. A 3 mm coronal everX-flow extension was made, and light-cured for 10 s (Fig. 5d-i and d-ii).

Following the reinforcement, molds were reassembled with the mold base and the clear index. Gradia core was injected through the index and light-cured for 10 s per surface. The index was then carefully removed, and all surfaces were re-cured (10 s per surface). Each index was used once and then discarded to avoid distortion. Excess core material was removed with a No.15 blade. Specimens were then finished and polished with SiC papers (600–2000 grit), using five gentle circular strokes per surface under water irrigation. Each specimen was carefully inspected under 3.5× magnification loupes, and any defective specimen was discarded.

Periodontal ligament simulation was not performed in this study to enhance reproducibility and isolate the variables of interest. Excluding this step reduced the risk of introducing additional variability that could compromise the reliability of static fracture testing outcomes [30, 31]. Finally, to ensure proper fit with the seating area of the universal testing machine (Instron 3365; Instron industrial products, Norwood, Massachusetts, USA), specimens were vertically embedded in auto-polymerizing acrylic resin blocks in cylindrical molds, exposing the core structure and 2 mm of the coronal mold structure.

### Fracture strength testing

**Fig. 6:**
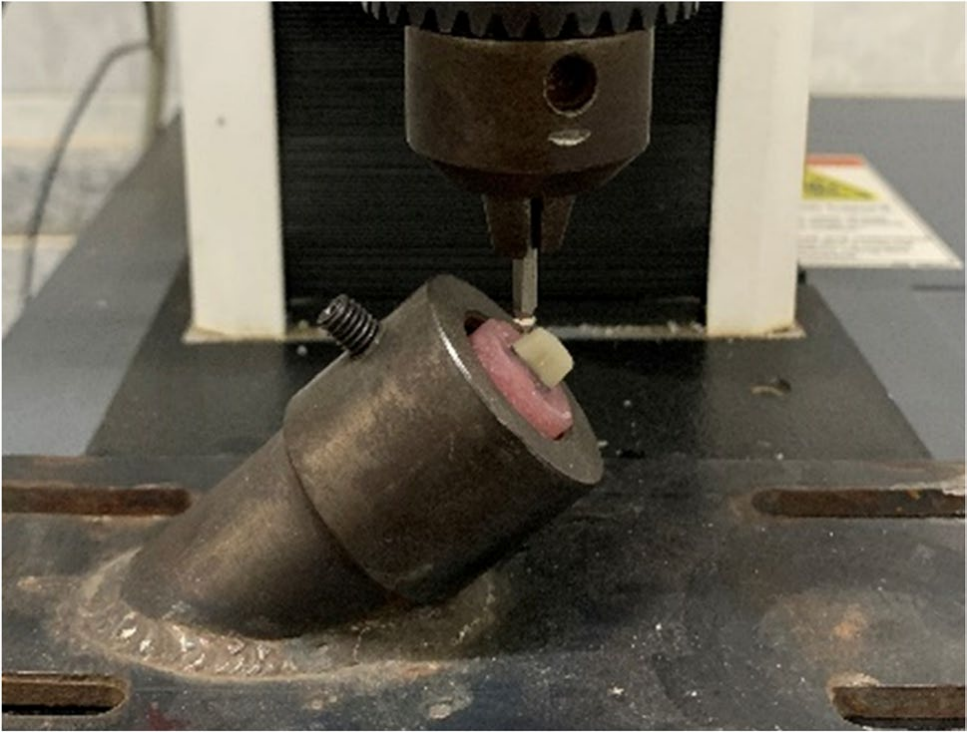
Fracture strength testing assembly

A universal testing machine was used to apply an increasing compressive load until fracture. Each specimen was firmly seated in the lower compartment using tightening screws. In the upper compartment, a 4 mm stainless steel spherical attachment was mounted. To simulate functional oblique forces commonly experienced by maxillary premolars, the load was applied at a 45° angle to the long axis of the specimen at a crosshead speed of 1 mm/min [32]. The point of loading application was standardized 2 mm from the occlusal edge of the palatal surface of the core buildup to approximate functional palatal cusp loading, concentrate shear stresses at the cervical/post interface, and prevent tipping during testing (Fig. 6). The maximum force at failure was recorded in Newtons (N). Fracture strength was identified visually and audibly and confirmed by a drop in the load-deflection curve [33].

### Failure mode analysis

**Fig. 7:**
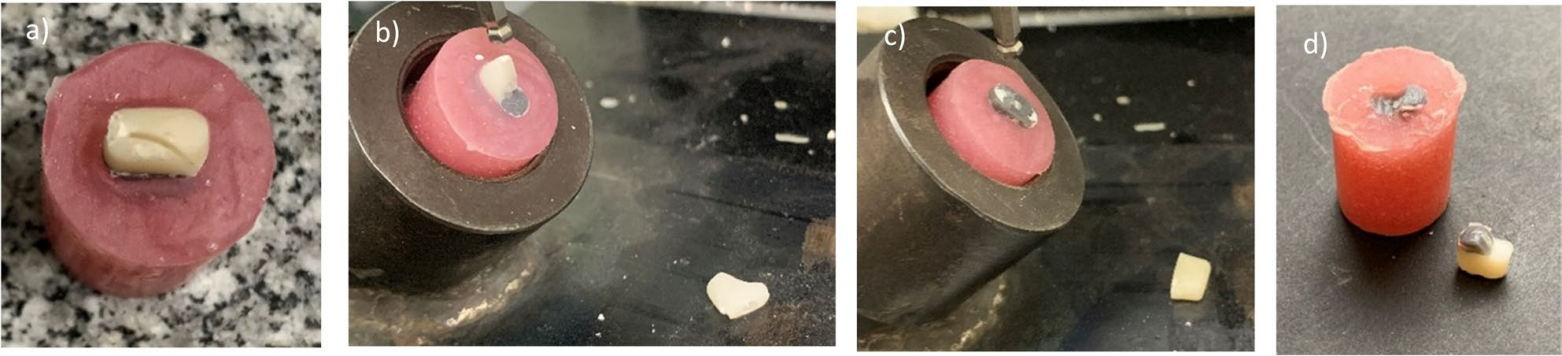
Different types of failure modes recorded during fracture strength testing (**a**) type I failure mode, (**b**) type II failure mode, (**c**) type III failure mode and (**d**) type IV failure mode

The failure mode of each specimen was visually examined and documented. Failures were classified into four types, adapted from previous literature [26, 34]:

*Type I (favorable)*- core fracture or detachment with no damage to the reinforcing material or root analogue (Fig. 7a).

*Type II (favorable)*- fracture involving the reinforcing material but confined above the acrylic embedment (Fig. 7b).

*Type III (favorable)*- fracture involving complete separation between the core, reinforcing material, and mold but confined above the acrylic embedment (Fig. 7c).

*Type IV (unfavorable)*- fracture extending to or beyond the acrylic resin, involving root analog fracture (Fig. 7d).

### Statistical analysis

Numerical data showed a normal distribution (*p* > 0.05, Shapiro-Wilk test) and homogeneity of variance (*p* > 0.05, Levene’s test). Mean ± standard deviation (SD) values were reported. A three-way ANOVA analyzed the main effects and interactions of simulated root canal morphology, PSD, and GFRC type on the fracture strength of post-core buildups. One-way ANOVA with Tukey’s HSD post hoc test evaluated the effect of simulated root canal morphology and GFRC type on fracture strength, regardless of PSD and within each PSD (4 mm and 6 mm). An independent samples *t*-test compared fracture strength between 4 mm and 6 mm PSD within each group.

Categorical data (failure mode) were described as frequency and percentage. Intergroup comparison of categorical variables was performed using the chi square test (Pa ≤ 0.0033). The significance level was set at α = 0.05. Adjusted standardized residuals were used to identify which cells contributed to the significant χ² results; absolute adjusted residuals ≳ 1.96 indicated cell counts differing from expected at α ≈ 0.05. Cramér’s V was reported as a measure of effect size for χ² results. Statistical analysis was performed using IBM^®^ SPSS version 22 (IBM; IBM Corp., Armonk, New York, USA).

## Results

A three-way ANOVA (Table 2) was conducted to examine the effects of simulated root canal morphology, PSD, and GFRC type on the fracture strength of post-core buildups. Only the main effect of GFRC type was statistically significant (F = 15.69, *p* < 0.001, partial η^2^ = 0.25), indicating that fracture strength differed significantly between materials. In contrast, neither simulated root canal morphology (F = 0.39, *p* = 0.68, partial η^2^ = 0.01), nor PSD (F = 0.51, *p* = 0.48, partial η^2^ = 0.003) had a significant effect. Statistically significant interaction was found between simulated root canal morphology x GFRC type (F = 2.24, *p* = 0.04, partial η^2^ = 0.09), indicating that the effect of GFRC type on fracture strength depend on the simulated root canal morphology. No statistically significant interactions were observed for simulated root canal morphology x PSD (F = 0.28, *p* = 0.76), PSD x GFRC type (F = 0.14, *p* = 0.94), or the three-way interaction (F = 0.41, *p* = 0.87) suggesting that these combinations did not produce significant effect on fracture strength.

**Table 2:** Three-way ANOVA of the effect of the different study variables and their interactions on fracture strength

Source	Type III Sum of Squares	df	Mean Square	F	*p*-value	Partial η²
Simulated root canal morphology	11989.12	2	5994.62	0.39	0.68	0.01
Post space depth	7675.00	1	7675.00	0.51	0.48	0.003
GFRC type	715486.65	3	238495.55	15.69	< 0.001*	0.25
Simulated root canal morphology x Post space depth	8346.92	2	4173.46	0.28	0.76	0.004
Simulated root canal morphology x GFRC type	204562.64	6	34093.77	2.24	0.04*	0.09
Post space depth x GFRC type	6183.42	3	2061.14	0.14	0.94	0.003
Simulated root canal morphology x Post space depth x GFRC type	37488.48	6	6248.08	0.41	0.87	0.02

*df * degree of freedom

Asterisks indicating significance (*p* < 0.05)

Regardless of PSD (Table 3), a statistically significant interaction was found between simulated root canal morphology and Itena post (*p* = 0.02). The double-circle canal analogues had the highest mean fracture strength, significantly greater than oval canal analogues. In contrast, everStick post, everX-flow and everX-posterior showed a no significant differences across the three simulated root canal morphologies (*p* = 0.15, 0.40, and 0.96 respectively).

In single-circle canal analogurs, there was no significant difference (*p* > 0.05) among Itena post, everStick post and everX-posterior, whereas everX-flow showed significantly lower fracture strength values compared to these materials. In the oval canal analogues, everStick post and everX-posterior showed significantly higher fracture strength values (*p* < 0.05) compared to everX-flow. In the double-circle canal analogues, Itena post showed significantly higher fracture strength values (*p* < 0.05) compared to everX-flow (Table 3).

At 4 mm PSD (Table 3), in single-circle canal analogues, everStick post and Itena post exhibited significantly higher fracture strength compared to everX-flow (*p* < 0.05). In oval canal analogues, everStick post exhibited significantly higher fracture strength compared to everX-flow (*p* < 0.05). In double-circle canal analogues, Itena post showed the highest mean fracture strength values, but differences among the four materials were not statistically significant (*p* > 0.05). No significant differences were found between simulated root canal morphologies within the same material (*p* > 0.05).

At 6 mm PSD (Table 3), in single-circle canal analogues, everX-flow showed significantly lower fracture strength than all the other materials (*p* < 0.05). No statistically significant differences were observed among the GFRCs in oval and double-circle canals (*p* > 0.05). Similarly, no significant differences were found between simulated canal morphologies within the same material (*p* > 0.05).

**Table 3:** Means ± SD in N for the effect of simulated root canal morphology within each GFRC type on fracture strength and the effect of GFRC type within each simulated root canal morphology, regardless the post space depth

GFRC type	Simulated root canal morphology			*p*-value
	Single-circle	Oval	Double-circle	
Itena post (control)	913.14 ± 103.86^bAB^	810.82 ± 120.13^abA^	941.38 ± 151.52^bB^	0.02*
everStick post	911.44 ± 104.26^bA^	907.41 ± 100.69^bA^	830.05 ± 152.70^abA^	0.15
everX-flow	698.76 ± 104.10^aA^	744.81 ± 135.48^aA^	753.94 ± 101.77^aA^	0.40
everX-posterior	880.56 ± 102.58^bA^	870.54 ± 116.05^bA^	881.15 ± 129.97^abA^	0.96
*p*- value	< 0.001*	0.004*	0.001*	

Means with the same lower-case letters within each column showed no statistically significant difference at *p* = 0.05

Means with same upper-case letters within each row showed no statistically significant difference at *p* = 0.05

Asterisks indicating significance (*p* = 0.05)

Independent samples t-test comparing fracture strength values between 4 mm and 6 mm PSD (Table 4) revealed no statistically significant difference across any root canal morphology or reinforcing material (*p* > 0.05).

**Table 4:** Means ± SD in N for the effect of each GFRC type within each simulated root canal morphology for 4 mm and 6 mm post space depth and the effect of post space depth within each GFRC type

Simulated root canal morphology	GFRC type	Post space depth		
		4 mm	6 mm	*p*-value
Single-circle	Itena post (control)	905.91 ± 117.44^b^	920.38 ± 97.26^b^	0.81
	everStick post	918.79 ± 140.81^b^	904.09 ± 59.99^b^	0.80
	everX flow	702.46 ± 101.95^a^	695.05 ± 114.25^a^	0.90
	everX posterior	851.43 ± 79.97^ab^	909.69 ± 120.09^b^	0.31
Oval	Itena post (control)	839.76 ± 104.71^ab^	781.87 ± 135.45^a^	0.39
	everStick post	911.41 ± 109.25^b^	903.41 ± 99.97 ^a^	0.89
	everX flow	727.09 ± 120.24^a^	762.52 ± 156.78^a^	0.64
	everX posterior	861.91 ± 85.90^ab^	879.17 ± 147.06^a^	0.79
Double-circle	Itena post (control)	922.99 ± 121.56^a^	959.78 ± 184.87^a^	0.67
	everStick post	789.36 ± 145.15^a^	870.74 ± 159.96^a^	0.34
	everX flow	757.46 ± 88.31^a^	750.43 ± 120.88^a^	0.90
	everX posterior	874.33 ± 154.19^a^	887.97 ± 112.77^a^	0.85

Means with the same lower-case letters within each column showed no statistically significant difference at *p* = 0.05

*p-*value represents an independent samples *t*-test between 4 mm and 6 mm

### Failure mode analysis

A chi-square test revealed no statistically significant association between simulated root canal morphology and failure mode (*X*^2^(6) = 4.09, *p* = 0.67, Cramér’s V = 0.11), indicating that no statistically significant association between simulated canal morphology and fracture mode.

**Fig. 8:**
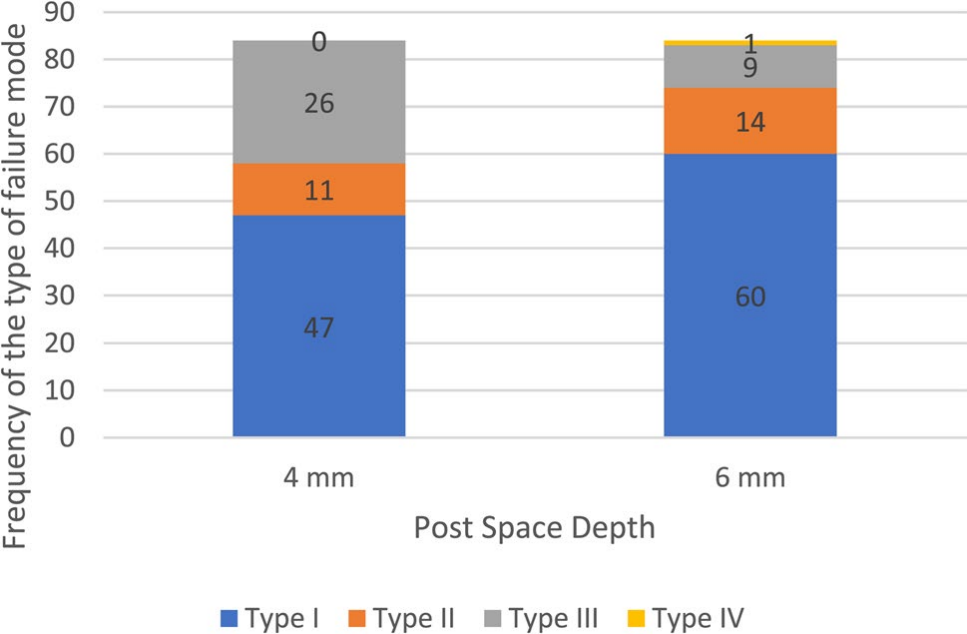
A stacked bar chart of frequency of failure modes according to the post space depths

In contrast, there was a statistically significant association between PSD and failure mode (*X*^2^(3) = 11.20, *p* = 0.01, Cramér’s V = 0.26), indicating that the PSD influences the mode of failure. Inspection of standardized residuals showed that Type I failures were observed more frequently than expected in the 6 mm PSD group (adjusted residual = + 2.1) while Type III failures were observed more frequently than expected in the 4 mm PSD group (adjusted residuals = + 3.2). Specimens of 4 mm PSD showed 56% type I failures, 13.1% type II, and 31% was of type III failure. In contrast, 6 mm PSD specimens exhibited 71.4% type I failure, 16.7% type II, 10.7% type III, and 1.2% type IV (Fig. 8).

**Fig. 9:**
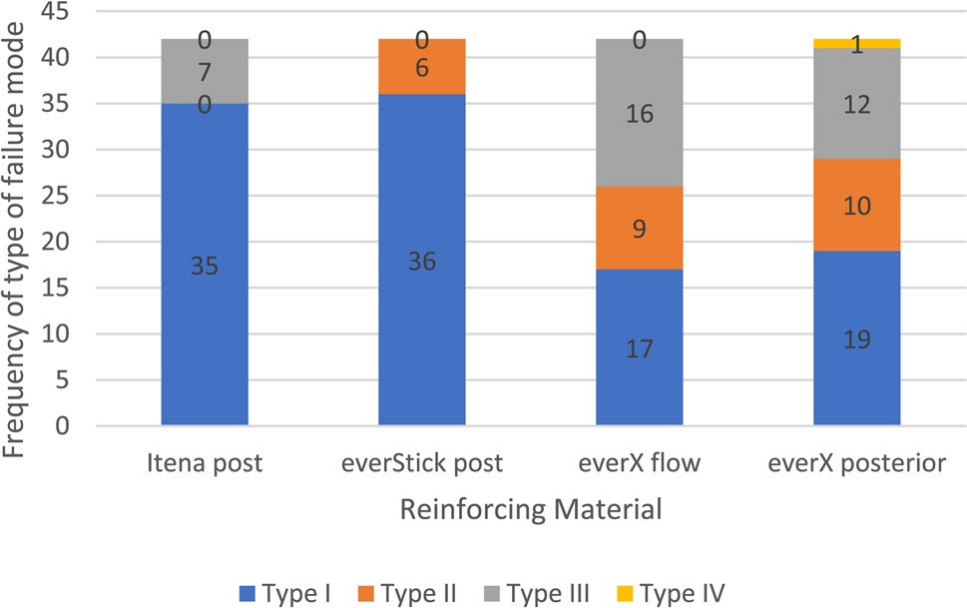
A stacked bar chart of frequency of failure modes according to the type of reinforcing material

A statistically significant association between reinforcing materials and failure mode was found (*X*^2^(9) = 40.58, *p* < 0.001, Cramér’s V = 0.28), indicating that the reinforcing material influences the mode of failure. Analysis of adjusted standardized residuals demonstrated that Itena and everStick posts significantly presented significantly more type I failures than expected (adjusted residuals = + 3.1 and + 3.4 respectively), whereas everX-flow and everX-posterior produced significantly fewer type I failures than expected (adjusted residuals = −3.6 and − 2.9 respectively). Conversely, everX-flow had a significantly greater than expected frequency of type III fractures (adjusted residuals = + 3.2). Only one type IV unfavorable failure occurred during this experimental study with everX-posterior group. Itena post specimens showed 83.3% type I and 16.7% type III failure modes. EverStick post specimens exhibited 85.7% type I and 14.3% type II failures. EverX-flow showed 40.5% type I failure, 21.4% type II failure, and 38.1% type III failure. EverX-posterior introduced type IV unfavorable failure of 2.4%, type I failure of 45.2%, 23.8% type II failure, and 28.6% type III failure (Fig. 9).

## Discussion

This *in vitro* study was designed to evaluate the effect of simulated root canal morphology (single-circle, oval, and double-circle), PSD (4 mm and 6 mm), and GFRC type (everStick post, everX-flow, and everX-posterior) on the fracture strength and failure mode of post-core buildups in 3D-printed root models of maxillary premolars. The prefabricated Itena post served as the control. The results revealed a dominant role for the GFRC type in both fracture strength and failure mode, along with a significant association between PSD and failure mode. In contrast, simulated root canal morphology exerted minimal statistical influence.

Based on the present study findings, the first null hypothesis was partially rejected, since GFRC type significantly influenced fracture strength (*p* < 0.001), while simulated root canal morphology and PSD did not. The second null hypothesis was also partially rejected, as both PSD and GFRC type were significantly associated with failure mode distributions (chi-square, *p* = 0.02, and *p <* 0.001 respectively), whereas simulated canal morphology was not. Collectively, these findings indicate that material selection plays a more decisive role than the remaining variables, in determining the mechanical performance and failure behavior of post-core systems under standardized *in vitro* conditions.

The significant difference observed among GFRC types and the control group can be attributed to their fundamentally different compositions, architectures, and physicomechanical properties [11, 35]. A significant interaction was also observed between simulated root canal morphology and GFRC type (*p* = 0.04), particularly in the case of Itena post. This finding maybe explained by the limited adaptability of prefabricated posts to irregular canal shapes, such as oval configurations, which may lead to thicker cement layers, voids entrapment and localized stress concentration [10, 13]. In contrast, individually formable everStick post, everX-flow, and everX-posterior performed consistently across all canal morphologies. This may be attributed to the everStick post superior anatomical adaptation and everX’s bulk fill behavior.

EverStick post, a pre-impregnated unidirectional E-glass fiber with interpenetrating polymer network (IPN) [36] and Itena post, a prefabricated unidirectional, parallel fiber system embedded in epoxy-resin, demonstrated higher fracture strength compared to everX-flow. The strength of everStick post may lie in the architecture of its high volume of continuous, parallel fibers embedded in a polymer resin matrix, creating a stiff, resilient structure that effectively and evenly distributes occlusal stresses along the post length and away from the cervical aspect of the root [37]. This stress dissipation mechanism, in addition to everStick post’s modulus of elasticity (~ 15 GPa), which approximates that of the dentin, may reduce the risk of unfavorable/non-repairable root fractures [38].

Comparable results were observed for Itena post across the different simulated canal morphologies and PSD. This may be attributed its modulus of elasticity, which is equivalent to that of dentin (~ 18–30 GPa) [38]. This elasticity enables stress distribution evenly along the root and minimize stress concentration points [38]. Although prefabricated, the Itena post design, alongside a compatible adhesive cement, enables adequate adaptation and load transfer [5, 38]. However, this may not hold true in oval, wide or flared canals, whereas everStick post offers superior canal conformity and mechanical performance due to its formability [39]. Although Itena post demonstrated slightly higher mean fracture strength than everStick post across simulated root canal morphology at both PSDs, the percentage differences were only 1.86% at 4 mm PSD and 0.6% at 6 mm PSD. These minimal differences suggest limited clinical significance, supporting a more conservative restorative approach.

EverX-posterior, a short fiber-reinforced composite composed of randomly oriented E-glass fibers within a semi-interpenetrating polymer network [40], also demonstrates comparable mean strength to everStick post and Itena post. The reinforcement mechanism of SFRCs differs fundamentally from that of fiber posts. The randomly oriented short E-glass fibers act as crack stoppers [40]. When a microcrack initiates and propagates through the resin, its energy is intercepted and blunted by the fibers, preventing further extension [41]. This significantly increases the material’s fracture toughness and load-bearing capacity. The high fiber loading of everX-posterior (74.2 wt%) contributes to its bulk strength and stiffness [42], making it a viable alternative to traditional post-core systems. The percentage differences between Itena post and everX-posterior were 3.08% at 4 mm PSD and 0.56% at 6 mm PSD, while the percentage differences between everStick post and everX-posterior were 1.22% at 4 mm PSD and 0.05% at 6 mm PSD.

In contrast, everX-flow demonstrated the lowest fracture strength across all morphological groups and PSDs. Although everX-flow employs the same short-fiber technology as everX-posterior, its lower fiber loading (25 wt%), different fiber geometry, random fiber orientation, flowable consistency, and the possibility of void entrapment during injection likely contributed to reduced mechanical properties and increased susceptibility to stress concentration, compared to its packable counterpart [43]. By contrast, everX-posterior’s longer and thicker fibers and high fiber loading offer greater resistance to compressive loading and crack initiation over a large area, making it suitable for high stress posterior restorations [44]. These findings underscore that while fiber reinforcement is beneficial, fiber volume fraction and density, along with the properties of the resin matrix, are critical determinants of the final restoration’s strength [45].

The present findings confirmed that neither simulated root canal morphology nor PSD demonstrated a statistically significant effect on fracture strength under the standardized conditions of this *in vitro *design. Although numerical variations were observed among subgroups, these differences were not sufficient to indicate a mechanical influence. This lack of significance may be attributed to the standardized geometries of the 3D-printed root analogues, the high adaptability and performance of modern reinforcing materials, and the adhesive interfaces that potentially homogenized stress distribution regardless of canal shape or depth.

Previous literature has suggested that irregular or widened canal shapes can compromise fracture resistance, particularly when prefabricated posts are used [5, 46, 47]; however, the present data did not reflect such differences statistically. This indicates that, when high-performance, formable GFRC systems are bonded under controlled conditions, canal shape alone does not appear to dictate mechanical outcomes. Likewise, the absence of a significant difference between 4 mm and 6 mm PSDs suggests that, within this limited range, post length was not a determining factor in fracture strength. Therefore, emphasis in both interpretation and clinical relevance has been placed on the material type, which was the only factor to produce statistically significant effects.

The failure mode analysis results showed that both GFRC type and PSD influence failure mode distribution in this *in vitro* model, whereas simulated canal morphology did not. The GFRC type effect was of medium magnitude (Cramer’s V = 0.28), and PSD produced a small to moderated effect (Cramer’s V = 0.26). Clinically, these findings suggest that the choice of intracanal reinforcing system and post length affect the failure pattern of restored teeth under oblique load; Itena and everStick posts produced disproportionately more Type I failures, while everX-flow and everX-posterior produced fewer type I and more type III failures. Similarly, 6 mm PSD was associated with a higher frequency of Type I outcomes, whereas 4 mm PSD was associated with more type III outcomes. These patterns suggest that in this *in vitro* experimental model, both the GFRC type properties and the degree of its intercanal extencion modulate the stress distribution and the mode of failure.

These results align partially with previous literature that reports material dependent differences in fracture behavior of fiber-reinforced restorations and mixed findings regarding post length. A 2023 system have reported that fiber-reinforced composites and fiber posts can modify both fracture strength and failure mode, although the magnitude and direction of effect depend on fiber architecture, resin matrix and continuity of post and core complex [11]. It was found that deeper intra-radicular reinforcement can be associated with improved load distribution and more favorable failure despite the excessive intra-radicular dentin preparation accompanied for post fitting [47], however reported inconsistent or non-significant effects of small changes in post length was observed in the literature [48]. This heterogeneity likely stems from differences in post design, tooth model, ferrule presence and test protocols.

This *in vitro* study was conducted under standardized laboratory conditions, which do not fully replicate the complex biological and mechanical environment of the oral cavity. The use of 3D-printed resin root analogues in place of natural dentin, although advantageous for standardization, does not reproduce the intrinsic properties of dentin such as moisture content, collagen structure, viscoelasticity, and fatigue behavior. In addition, no periodontal ligament simulation was provided in the specimen design, which may influence stress dissipation patterns under functional loading. Another limitation is the absence of a ferrule or full-coverage crown, both of which are known to significantly enhance the fracture strength of ETT and alter failure patterns. Furthermore, the static load-to-fracture protocol does not account for thermomechanical aging, cyclic fatigue, or long-term degradation of materials. Furthermore, the absence of oral environmental factors that are known to cause deterioration of resins specially fiber-reinforced ones as moisture, pH and thermal fluctuations, cyclic fatigue and bacterial activity is a present study limitation. Therefore, while the current findings allow mechanical comparisons among GFRC types, PSDs, and simulated canal morphologies under controlled conditions, caution should be exercised when extrapolating these results to clinical scenarios. Future studies incorporating ferrule design, dynamic loading, thermocycling, and natural tooth substrates are needed to validate and extend these outcomes.

## Conclusion

Within the limitations of this *in vitro* study, it can be concluded that the selection of the intracanal reinforcing material is the most crucial factor in determining the fracture performance of post-core buildups than root canal morphology and post space depth. The mechanical reinforcement of everStick and everX-posterior posts is comparable to that of Itena posts.

### Clinical implications

These results support the following recommendations for clinical practice:

- The type of reinforcing material should take precedence over post depth or canal type in reinforcing ETT.
- EverStick post and everX-posterior can replace Itena post as a conservative reinforcing treatment option in single-canaled premolars.
- Shorter post space depth (4 mm) can be sufficient, especially when combined with glass fiber-reinforced materials.

## Data Availability

The datasets used and/or analyzed during the current study are available from the corresponding author on reasonable request.

## References

[1] Patel S, Bhuva B, Bose R. Present status and future directions: vertical root fractures in root filled teeth. Int Endod J. 2022;55(2):804–26. 10.1111/iej.13737. 35338655 10.1111/iej.13737 PMC9324143

[2] Laculli F, Rengo C, Lodato V, Patini R, Spagnuolo G, Rengo S. Fracture resistance of endodontically-treated maxillary premolars restored with different type of posts and direct composite reconstructions: a systematic review and meta-analysis of in vitro studies. Dent Mater. 2021;37(9):e455-84. 10.1016/j.dental.2021.06.007. 34148785 10.1016/j.dental.2021.06.007

[3] Caussin E, Izart M, Ceinos R, Attal J, Beres F, François P. Advanced material strategy for restoring damaged endodontically treated teeth: a comprehensive review. Materials. 2024;17(15):3736. 10.3390/ma17153736. 39124400 10.3390/ma17153736 PMC11313123

[4] Karobari M, Veeraraghavan V, Nagarathna P, Varma S, Kodangattil Narayanan J, Patil S. Predictive analysis of root canal morphology in relation to root canal treatment failures: a retrospective study. Frontiers in Dental Medicine. 2025;6:1540038. 10.3389/fdmed.2025.1540038. 40343090 10.3389/fdmed.2025.1540038 PMC12058801

[5] Webber MBF, Bernardon P, França FMG, Amaral FLB, Basting RT, Turssi CP. Oval versus circular-shaped root canals: bond strength reached with varying post techniques. Braz Dent J. 2018;29(4):335–41. 10.1590/0103-6440201801937. 30462758 10.1590/0103-6440201801937

[6] Tsukahara R, Komada W, Oishi S, Yoshimatsu S, Miura H, Fueki K. Fracture strength of flared root canals reinforced using different post and core materials. J Prosthodont. 2023;32(7):639–45. 10.1111/jopr.13616. 36270777 10.1111/jopr.13616

[7] Shaikh SY, Shaikh SS. Direct linear measurement of root dentin thickness and dentin volume changes with post space preparation: a cone-beam computed tomography study. Contemp Clin Dent. 2018;9(1):77–82. 10.4103/ccd.ccd_785_17. 29599589 10.4103/ccd.ccd_785_17 PMC5863415

[8] Kishen A, Kumar G, Chen N. Stress-strain response in human dentine: rethinking fracture predilection in postcore restored teeth. Dent Traumatol. 2004;20(2):90–100. 10.1111/j.1600-4469.2004.00250.x. 15025691 10.1111/j.1600-4469.2004.00250.x

[9] Chuang SF, Yaman P, Herrero A, Dennison JB, Chang CH. Influence of post material and length on endodontically treated incisors: an in vitro and finite element study. J Prosthet Dent. 2010;104(6):379–88. 10.1016/S0022-3913(10)60171-0. 21095401 10.1016/S0022-3913(10)60171-0

[10] Bhaktikamala A, Chengprapakorn W, Serichetaphongse P. Effect of different post materials and adaptability on fracture resistance and fracture mode in human endodontically treated teeth. Int J Dent. 2022;9170081. 10.1155/2022/9170081. 35966224 10.1155/2022/9170081 PMC9371825

[11] Selvaraj H, Krithikadatta J, Shrivastava D, Onazi M, Algarni H, Munaga S, et al. Systematic review fracture resistance of endodontically treated posterior teeth restored with fiber reinforced composites- a systematic review. BMC Oral Health. 2023;23(1):566. 10.1186/s12903-023-03217-2. 37574536 10.1186/s12903-023-03217-2 PMC10423428

[12] Fousekis E, Lolis A, Marinakis E, Oikonomou E, Foros P, Koletsi D, et al. Short fiber-reinforced composite resins as post-and-core materials for endodontically treated teeth: a systematic review and meta-analysis of in vitro studies. J Prosthet Dent. 2025;134(1):61–71. 10.1016/j.prosdent.2023.09.026. 37919126 10.1016/j.prosdent.2023.09.026

[13] de Morais D, Butler S, Santos M. Current insights on fiber posts: a narrative review of laboratory and clinical studies. Dent J (Basel). 2023;11(10):236. 10.3390/dj11100236. 37886921 10.3390/dj11100236 PMC10605739

[14] Huang M, Wang B, Zhang K, Yan X, Chen Z, Zhang X. Comparative analysis of stress distribution in residual roots with different canal morphologies: evaluating CAD/CAM glass fiber and other post-core materials. BMC Oral Health. 2024;24(1):337. 10.1186/s12903-024-04109-9. 38491485 10.1186/s12903-024-04109-9 PMC10943834

[15] Cohen J. Statistical power analysis for the behavioral sciences. 2nd ed. Hillsdale: Lawrence Erlbaum Associates; 1988.

[16] Ghorayeb SR, Valle T. Experimental evaluation of human teeth using noninvasive ultrasound: echodentography. IEEE Trans Ultrason Ferroelectr Freq Control. 2002;49(10):1437–43. 10.1109/TUFFC.2002.1041085. 12403145 10.1109/tuffc.2002.1041085

[17] Saber SEDM, Ahmed MHM, Obeid M, Ahmed HMA. Root and canal morphology of maxillary premolar teeth in an Egyptian subpopulation using two classification systems: a cone beam computed tomography study. Int Endod J. 2019;52(3):267–78. 10.1111/iej.13016. 30225932 10.1111/iej.13016

[18] Liu X, Gao M, Ruan J, Lu Q. Root canal anatomy of maxillary first premolar by microscopic computed tomography in a Chinese adolescent subpopulation. Biomed Res Int. 2019;4327046. 10.1155/2019/4327046. 31828103 10.1155/2019/4327046 PMC6881762

[19] Li YH, Bao SJ, Yang XW, Tian XM, Wei B, Zheng YL. Symmetry of root anatomy and root canal morphology in maxillary premolars analyzed using cone-beam computed tomography. Archof Oral Biol. 2018;94:84–92. 10.1016/j.archoralbio.2018.06.020. 10.1016/j.archoralbio.2018.06.020 29990589

[20] Ferraro JM, Falter J, Lee S, Watanabe K, Wu TH, Kim DG, et al. Accuracy of three-dimensional printed models derived from cone-beam computed tomography. Angle Orthod. 2022;92(6):722–7. 10.2319/021122-128.1. 35852459 10.2319/021122-128.1 PMC9598849

[21] Koç S, Kırmalı Ö, Çelik HK. Evaluation of stress patterns in teeth with endodontic treatment and periapical lesions as abutments for fixed prosthesis: a finite element analysis study. BMC Oral Health. 2025;25(1):130. 10.1186/s12903-025-05501-9. 39856673 10.1186/s12903-025-05501-9 PMC11760115

[22] Muñoz C, Llena C, Forner L. Oval fiber posts do not improve adaptation to oval-shaped canal walls. J Endod. 2011;37(10):1386–9. 10.1016/j.joen.2011.07.003. 21924187 10.1016/j.joen.2011.07.003

[23] Formlab. (2023) Rigid 10K Resin Technical Data Sheet. https://formlabs-media.formlabs.com › datasheets › 2001479. Accessed 25 Jun 2025.

[24] Kinney JH, Marshall SJ, Marshall GW. The mechanical properties of human dentin: a critical review and re-evaluation of the dental literature. Crit Rev Oral Biol Med. 2003;14(1):13–29. 10.1177/154411130301400103. 12764017 10.1177/154411130301400103

[25] Craig R, Powers JM. Restorative Dental Materials. 13 ed. Mosby, Elsevier Inc.; 20120.

[26] Aboelnor MM, Nour KA, Al-Sanafawy HMA. Fracture strength of direct occlusal veneers with different short fiber-reinforced composite cores and veneering materials: an in-vitro study. Clin Oral Investig. 2024;28(12):635. 10.1007/s00784-024-06013-6. 39523240 10.1007/s00784-024-06013-6 PMC11551077

[27] Oh WS, Kim SE. Modification of interim removable partial denture using thermoplastic vacuum-formed matrix. J Prosthet Dent. 2008;99(6):494–6. 10.1016/S0022-3913(08)60116-X. 18514674 10.1016/S0022-3913(08)60116-X

[28] Mehta SB, Banerji S, Millar BJ, Suarez-Feito JM. Current concepts on the management of tooth wear: part 4. An overview of the restorative techniques and dental materials commonly applied for the management of tooth wear. Br Dent J. 2012;212(4):169–77. 10.1038/sj.bdj.2012.137. 22361546 10.1038/sj.bdj.2012.137

[29] ADA/FDI. (2021) How to avoid common failures in adhesive den tistry. PracticeUpdate. https://www. pract iceup date. com/ conte nt/ adafdi- 2021- how- to- avoid- common- failu res- in- adhes ive- denti stry/125188. Accessed 1 Jul 2025.

[30] Nawafleh N, Bibars AR, Elshiyab S, Janzeer Y. In vitro simulation of periodontal ligament in fatigue testing of dental crowns. Eur J Dent. 2020;14(3):380–5. 10.1055/s-0040-1713953. 32645731 10.1055/s-0040-1713953 PMC7440937

[31] Marchionatti AM, Wandscher VF, Broch J, Bergoli CD, Maier J, Valandro LF, Kaizer OB. Influence of periodontal ligament simulation on bond strength and fracture resistance of roots restored with fiber posts. J Appl Oral Sci. 2014;22(5):450–8. 10.1590/1678-775720140067. 25466478 10.1590/1678-775720140067 PMC4245758

[32] Sharma A, Amirtharaj LV, Sanjeev K, Mahalaxmi S. Fracture resistance of endodontically treated premolars restored with flowable short fibre-reinforced resin composite-an in vitro study. Eur Endod J. 2022;7(2):161–6. 10.14744/eej.2021.07830. 35786574 10.14744/eej.2021.07830 PMC9285990

[33] Al-Ibraheemi ZA, Abdullah HA, Jawad NA, Haider J. Assessing fracture resistance of restored premolars with novel composite materials: an in vitro study. Int J Dent. 2021;5512708. 10.1155/2021/5512708. 34462637 10.1155/2021/5512708 PMC8403034

[34] Piccioli F. Fracture resistance of endodontically treated premolars restored with direct fiberglass-reinforced composite in MOD cavities. J Clin Dentistry Oral Health. 2019;3(2):1–5. 10.35841/oral-health.3.2.1-5.

[35] Kumar AL, Prakash M. The effect of fiber orientation on mechanical properties and machinability of GFRP composites by end milling using cutting force analysis. Polym Polym Compos. 2021;29(9_suppl):S178–87. 10.1177/0967391121991289.

[36] Khurana D, Prasad AB, Raisingani D, Srivastava H, Mital P, Somani N. Comparison of ribbond and Everstick post in reinforcing the re-attached maxillary incisors having two oblique fracture patterns: an in vitro study. Int J Clin Pediatr Dent. 2021;14(5):689–92. 10.5005/jp-journals-10005-2035. 34934284 10.5005/jp-journals-10005-2035 PMC8645633

[37] Deepa VL, Reddy SN, Garapati VC, Sudhamashetty SR, Yadla P. Fracture fragment reattachment using projectors and anatomic Everstick Post™: an ultraconservative approach. J Int Soc Prev Community Dent. 2017;7(Suppl 1):S52-4. 10.4103/jispcd.JISPCD_151_17. 28713769 10.4103/jispcd.JISPCD_151_17 PMC5502553

[38] de Morais DC, Butler S, Santos MJMC. Current insights on fiber posts: a narrative review of laboratory and clinical studies. Dent J. 2023;11(10):236. 10.3390/dj11100236. 10.3390/dj11100236 PMC10605739 37886921

[39] Beltagy TM. Fracture resistance of rehabilitated flared root canals with anatomically adjustable fiber post. Tanta Dent J. 2017;14(2):96–103. 10.4103/tdj_16_17.

[40] Garoushi S, Gargoum A, Vallittu PK, Lassila L. Short fiber-reinforced composite restorations: a review of the current literature. J Investig Clin Dent. 2018;9(3):e12330. 10.1111/jicd.12330. 29479830 10.1111/jicd.12330

[41] Bijelic-Donova J, Garoushi S, Lassila LV, Rocca GT, Vallittu PK. Crack propagation and toughening mechanism of bilayered short-fiber reinforced resin composite structure -evaluation up to six months storage in water. Dent Mater J. 2022;41(4):580–8. 10.4012/dmj.2021-321. 35584936 10.4012/dmj.2021-321

[42] Alshabib A, Jurado CA, Tsujimoto A. Short fiber-reinforced resin-based composites (SFRCs); current status and future perspectives. Dent Mater J. 2022;41(5):647–54. 10.4012/dmj.2022-080. 35858793 10.4012/dmj.2022-080

[43] Youssef HH, Abed YA, ElZoghbi AMF, Negm HMH. Evaluation of fracture resistance of fiber-reinforced resin composie restortions in deep class I cavities: a comparative in vitro study. Ain Shams Dent J. 2024;35(3):199–210. 10.21608/asdj.2024.317366.1487.

[44] Garoushi S, Säilynoja E, Frater M, Keulemans F, et al. A comparative evaluation of commercially available short fiber-reinforced composites. BMC Oral Health. 2024;24:1573. 10.1186/s12903-024-05267-6. 39736654 10.1186/s12903-024-05267-6 PMC11684104

[45] Mudunuri S, Varma K, Satish R, Kumar M, Dinesh J, Kumar P. Fiber-reinforced composites in endodontic practice: a review. Int J Dent Mater. 2020;2(4):122–34. 10.37983/IIDM.2020.2404.

[46] Versiani MA, Pécora JD, de Sousa-Neto MD. Flat-oval root canal preparation with self-adjusting file instrument: a micro-computed tomography study. J Endod. 2011;37(7):1002–7. 10.1016/j.joen.2011.03.017. 21689560 10.1016/j.joen.2011.03.017

[47] Marinescu AG, Abuabboud O, Zimbru ȘD, Cîrligeriu LE, Piț BA, Borcean IA, et al. Influence of the fiber post length on the fracture strength of endodontically treated teeth. Medicina (Kaunas). 2023;59(10):1797. 10.3390/medicina59101797. 37893515 10.3390/medicina59101797 PMC10608114

[48] Junqueira RB, de Carvalho RF, Marinho CC, Valera MC, Carvalho CAT. Influence of glass fibre post length and remaining dentine thickness on the fracture resistance of root filled teeth. Int Endod J. 2017;50(6):569–77. 10.1111/iej.12653. 27101091 10.1111/iej.12653

